# Interleukin-7 receptor blockade suppresses adaptive and innate inflammatory responses in experimental colitis

**DOI:** 10.1186/1476-9255-9-39

**Published:** 2012-10-12

**Authors:** Cynthia R Willis, Audrey Seamons, Joe Maxwell, Piper M Treuting, Laurel Nelson, Guang Chen, Susan Phelps, Carole L Smith, Thea Brabb, Brian M Iritani, Lillian Maggio-Price

**Affiliations:** 1 Department of Inflammation, Amgen, Inc, Seattle, WA, USA; 2 Department of Comparative Medicine, University of Washington, Seattle, WA, USA; 3 Fred Hutchinson Cancer Research Center, Seattle, WA, USA

**Keywords:** IL-7R, Colitis, *Mdr1a*^−/−^ mice, *Rag2*^−/−^ mice, *Helicobacter bilis*

## Abstract

**Background:**

Interleukin-7 (IL-7) acts primarily on T cells to promote their differentiation, survival, and homeostasis. Under disease conditions, IL-7 mediates inflammation through several mechanisms and cell types. In humans, IL-7 and its receptor (IL-7R) are increased in diseases characterized by inflammation such as atherosclerosis, rheumatoid arthritis, psoriasis, multiple sclerosis, and inflammatory bowel disease. In mice, overexpression of IL-7 results in chronic colitis, and T-cell adoptive transfer studies suggest that memory T cells expressing high amounts of IL-7R drive colitis and are maintained and expanded with IL-7. The studies presented here were undertaken to better understand the contribution of IL-7R in inflammatory bowel disease in which colitis was induced with a bacterial trigger rather than with adoptive transfer.

**Methods:**

We examined the contribution of IL-7R on inflammation and disease development in two models of experimental colitis: *Helicobacter bilis* (*Hb*)-induced colitis in immune-sufficient *Mdr1a*^−/−^ mice and in T- and B-cell-deficient *Rag2*^−/−^ mice. We used pharmacological blockade of IL-7R to understand the mechanisms involved in IL-7R-mediated inflammatory bowel disease by analyzing immune cell profiles, circulating and colon proteins, and colon gene expression.

**Results:**

Treatment of mice with an anti-IL-7R antibody was effective in reducing colitis in *Hb*-infected *Mdr1a*^−/−^ mice by reducing T-cell numbers as well as T-cell function. Down regulation of the innate immune response was also detected in *Hb*-infected *Mdr1a*^−/−^ mice treated with an anti-IL-7R antibody. In *Rag2*^−/−^ mice where colitis was triggered by *Hb*-infection, treatment with an anti-IL-7R antibody controlled innate inflammatory responses by reducing macrophage and dendritic cell numbers and their activity.

**Conclusions:**

Results from our studies showed that inhibition of IL-7R successfully ameliorated inflammation and disease development in *Hb*-infected mice by controlling the expansion of multiple leukocyte populations, as well as the activity of these immune cells. Our findings demonstrate an important function of IL-7R-driven immunity in experimental colitis and indicate that the therapeutic efficacy of IL-7R blockade involves affecting both adaptive and innate immunity.

## Background

Inflammatory bowel disease (IBD) is characterized by chronic and relapsing inflammation of the gastrointestinal tract. In humans, Crohn’s disease and ulcerative colitis are driven by a complex interplay between genetic and environmental factors, which contribute to chronic inflammation. Mouse models of colitis have been useful in determining the contributions of specific adaptive and innate immune mechanisms involved in the pathogenesis of IBD [1]. Experimental studies in mice have revealed that a combination of genetic factors, diet, and immune responses to microbial organisms can contribute to IBD susceptibility.

In both humans and mouse models, several lines of evidence suggest a significant role for IL-7 in inflammatory diseases, including IBD. Firstly, under normal conditions, local IL-7 production acts on resident T cells through IL-7 receptor (IL-7R; a heterodimer composed of IL-7Rα and IL-2Rγ chains) to promote their differentiation, survival and homeostasis [2]. IL-7 is produced by stromal cells and intestinal epithelial cells, suggesting a role for IL-7 in modulating immune responses in the intestinal microenvironment. Secondly, elevated serum concentrations of IL-7 have been detected in patients with ulcerative colitis [3] and Crohn’s disease [4], and gastric tissue biopsies from patients infected with *H*. *pylori* have increased IL-7 message [5]. Polymorphisms in *IL7RA* are also associated with several inflammatory diseases including ulcerative colitis [6]. Thirdly, the importance of IL-7 as a mediator of intestinal inflammation has been demonstrated in IL-7 transgenic mice which develop colitis resembling ulcerative colitis [7], and in IL-7 deficient mice, which are resistant to the development of non-T-, non-B-cell-mediated colitis [8]. Finally, the importance of IL-7-responsive-T cells in colitis has been demonstrated in TCRα^−/−^ mice, which develop spontaneous colitis driven by IL-7R^high^ memory T cells [9,10]. Adoptive transfer studies in these mice suggest that the colitogenic T cells are primarily memory CD4^+^ cells which express high IL-7Rα and are maintained and expanded with IL-7 [9,10], indicating that IL-7 signaling is important in IBD pathogenesis. In an adoptive T-cell-transfer model of colitis, Yamazaki and colleagues successfully treated ongoing colitis using a saporin-conjugated anti-IL-7Rα antibody, selectively eliminating lamina propria lymphocytes (LPL) with high expression of IL-7R [10]. These and other studies suggest that therapies interfering with IL-7R signaling could abrogate intestinal inflammation in IBD.

The aim of this study was to determine whether inhibition of IL-7Rα signaling would ameliorate colon inflammation induced by a bacterial trigger rather than with adoptive transfer of T cells. We used *Helicobacter bilis* (*Hb*) infection to induce colitis in T-cell-sufficient (multiple drug resistance 1a null; *Mdr1a*^−/−^) mice, and T- and B-cell-deficient (recombination activating gene 2; *Rag2*^−/−^) mice. This method has the advantage of not requiring adoptive transfer of T cells into immunodeficient hosts, which requires IL-7 for T-cell expansion and survival. In addition, our colitis models provided the opportunity to assess the cell types and functions affected by blocking IL-7Rα in mice where intestinal immunopathology is associated with T cells (in *Mdr1a*^−/−^ mice), and innate immune cells (in *Rag2*^−/−^ mice). Our results indicated that pharmacological inhibition of IL-7Rα reduced inflammation and subsequent disease development in *Hb*-infected mice by controlling the expansion of multiple leukocyte populations, as well as the activity of these immune cells. Our findings demonstrate an important function of IL-7R-driven immunity in experimental colitis involving both adaptive and innate immunity.

## Methods

### Mice, induction of colitis, and antibody treatments

Specific-pathogen free, 4–11 week old *Mdr1a*^−/−^ mice (FVB.129P2-*Abcb1a*^*tm1Bor*^, Taconic Farms, Albany, NY) [11,12]; bred in house), and *Rag2*^−/−^ mice (129S6/SvEvTac-Rag2) were verified to be free of *Helicobacter* species by fecal PCR [13]. Mice were treated by intraperitoneal (IP) injection with anti-IL-7Rα M595 (rat IgG2b; t_1/2_ = 3 days; Amgen Inc.) or isotype control M495 (rat IgG2b; Amgen Inc.) antibodies twice weekly, one week prior to oral gavage with *Hb* and continuing for the study duration (total of 10 weeks for *Mdr1a*^−/−^ mice and 12 weeks for *Rag2*^−/−^ mice). *Hb*-infection was confirmed by fecal PCR [13]. Mice were weighed weekly and euthanized when they developed 20% body-weight loss or signs of severe IBD, and tissue samples were taken. All mice were used according to procedures approved by Amgen’s and UW’s Animal Care and Use Committees.

### Pathology and immunohistochemistry

Necropsy, tissue sampling, processing and histologic examination was performed as previously described [11]. Cecum and colon IBD scores were based on severity of mucosal epithelial changes, degree of inflammation and extent of lesions [11]. 4 μm sections of formalin fixed, paraffin embedded cecum and colon were stained with biotinylated F4/80 antibody (clone CI:a3-1;AbD Serotec, Raleigh, NC) or isotype control, followed by development with horseradish peroxidase-labeled streptavidin and diaminobenzidine substrate solution. F4/80^+^ cells were scored based on numbers of cells with membrane staining as follows: 0 = no staining, 1 = minimal to few faintly positive cells, 2 = scattered single positive cells, 3 = clusters of two or more positive cells, 4 = larger clusters of positive cells, multifocal to coalescing. The mucosa, lamina propria, submucosa and tunica muscularis/serosa of both cecum and colon were scored and summed (maximum score = 32).

### Flow cytometric analysis

Single cell suspensions were made from spleen and MLN harvested at necropsy [14]. Cellularity was determined using a hemocytometer or with an Advia 120 Hematology Analyzer (Siemens, Deerfield, IL). Cells were blocked with anti-CD16/CD32 (BD Biosciences, San Jose, CA) then stained with fluorescein isothiocyanate-, phycoerythrin-, peridinin-chlorophyll-protein complex-Cy5.5-, or allophycocyanin-labeled antibodies (BD) to the following surface markers: anti-CD44, CD4, CD8a, CD62L, CD127 (clone B12-1 binds a different epitope than 5 anti-IL-7Rα M59), CD49D (clone DX5), CD11b, and CD11c. Data were collected on BD’s FACSCalibur or LSRII and analyzed using FlowJo (Tree Star, Inc, Ashland, OR).

### Serum cytokines and anti-*Hb* antibodies

Serum obtained at necropsy was analyzed using the Rodent MAP version 2.0 antigen panel (RBM, Austin, TX). Serum *Hb*-specific IgG2a was determined by ELISA as previously described [11] with the exception of using HRP-conjugated anti-mouse IgG2a (Southern Biotech, Birmingham, AL).

### Colonic-explant culture cytokines

5 mm of proximal colon was flushed with PBS containing penicillin/streptomycin, blotted on sterile gauze and then cultured in RPMI supplemented with 10% FCS, 1X non-essential amino acids (Irvine Scientific, Santa Ana, CA), 20 mM Hepes, 100 U/ml Penicillin, 100 ug/ml Streptomycin, 50 μM 2-mercaptoethanol, 2 mM L-glutamine, 1 mM sodium pyruvate at 37°C and 5% CO_2_ for 24 hours. Supernatants were harvested, clarified by centrifugation, and analytes were detected using the LINCOplex Mouse Cytokine/Chemokine Panel-32 plex (Millipore, Billerica, MA) and a Luminex 100 IS (Luminex, Austin, TX). Data were analyzed using the Data Interpretation Application (Luminex). Values were corrected for the weight of the tissue and are presented as concentration of protein/mg tissue in the cultures.

### Quantitative RT-PCR analyses

RNA was extracted from 5 mm of proximal colon (flushed with PBS) using the RNeasy kit (Qiagen, Valencia, CA). RNA was converted to cDNA using the High Capacity cDNA Reverse Transcription Kit (Applied Biosystems, Foster City, CA). Gene expression (relative to HPRT) was determined using TaqMan^®^ Mouse Immune Array (Applied Biosystems). Duplicate 150 μg cDNA samples were loaded per reservoir. Data were collected on an ABI 7900 LDA and analyzed using SDS v2.2.2, Spotfire DecisionSite v19.3.1006 (TIBCO Software Inc., Somerville, MA), Microsoft Excel 2003, and GraphPad Prism 5. IL-7Rα expression (relative to Gapdh) was determined on an Mx3005P (Stratagene, La Jolla, CA) using Power SYBR Green PCR Master Mix (Applied Biosystems). Samples were run in duplicate. Primer sequences were as follows: IL-7Rα-forward: 5^′^-ACAAGAACAACAATCCCACAGAG, IL-7Rα-reverse: 5^′^-TCGCTCCAGAAGCCTTTGAAG Gapdh forward: 5^′^-TTCCGTGTTCCTACCCCCAATGTG, and Gapdh reverse: 5^′^-TAGCCCAAGATGCCCTTCAGTG.

### Statistical methods

The Analysis of Variance (ANOVA) method was used to determine differences between treatment groups in colon mRNA expression, colon and serum protein panels, and FACS data. Comparisons between individual treatment groups were then performed with multiple comparison adjustment using either Tukey, Dunnett, or multivariate t methods. Homogeneous variance and normality assumptions were tested using Brown-Forsythe’s test and Shapiro-Wilk test, respectively. If either test showed a significant result (p < 0.01), a logarithm (logarithm base 2 for mRNA data) or square root transformation was considered. The transformation that satisfied both assumption tests was applied to the data. If both transformations failed, nonparametric ANOVA was used. The analyses were performed using SAS^©^ 9.2 (SAS Institute Inc., Cary, NC). Heat maps were generated using Spotfire DecisionSite software. For IBD scores, a similar analysis approach was applied using Graphpad Prism 5 software. An ANOVA analysis was performed. A square root transformation was applied to correct heterogeneous variance among groups. Tukey's multiple comparison test was applied for group comparisons.

## Results

### Blockade of IL-7Rα ameliorated *Hb*-induced colitis in *Mdr1a*^−/−^ mice

**Figure 1:**
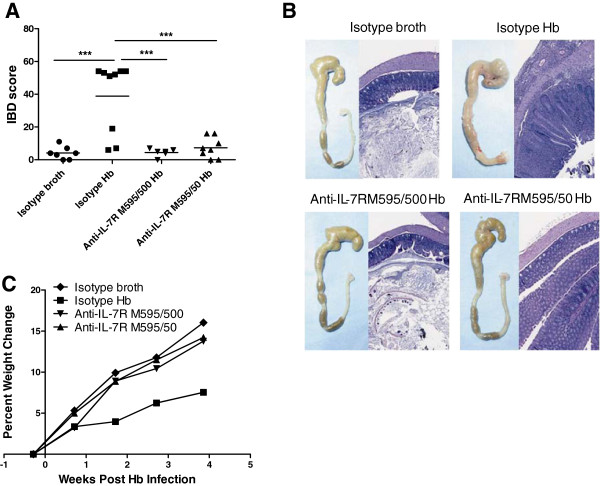
**Anti-IL-7Rα antibody treatment reduced IBD scores and histopathology of *H*. *bilis*-induced colitis in *Mdr1a***^**−/−**^**mice.** Five-week-old male *Mdr1a*^−/−^ mice were treated IP twice weekly with 500 μg or 50 μg of anti-IL-7Rα M595, or 500 μg of isotype control antibody one week prior to oral gavage with *H*. *bilis* (*Hb*, 2 × 10^7^ CFU administered once) or broth (uninfected controls) and then treated twice weekly throughout the duration of the study for 9 weeks post-infection. (**A**) Histopathological IBD scores from colon. Cecum and colon IBD scores were based on severity of mucosal epithelial changes, degree of inflammation and extent of lesions [11]. (**B**) Gross and representative H&E-stained histological sections from uninfected control (isotype broth), infected control (isotype *Hb*) and anti-IL-7Rα M595/500 and anti-IL-7Rα M595/50-treated *Hb*-infected mice. Histological images, original magnification 10X. (**C**) Percent change in weight is shown for mice 1 week prior to infection through 4 weeks post infection (last time point in which all mice were alive). ANOVA with square root transformation followed by Tukey’s post-test (a). *p < 0.05, **p < 0.001, ***p <0.0001.

*Mdr1a*^−/−^ mice develop colitis when infected with certain enteric helicobacter species [11]. To determine the effectiveness of blocking IL-7Rα in colitis, we administered an anti-IL-7Rα antibody to *Mdr1a*^−/−^ mice beginning one week prior to infection with *Hb* and continuing for the duration of the experiment. Previous studies with anti-IL-7Rα antibodies resulted in decreased lymphocyte numbers in naïve and diseased mice (Amgen Inc.; data not shown). Therefore, we used two doses of anti-IL-7Rα M595 to determine if a 10-fold lower dose would preserve lymphocyte numbers yet still afford protection from colitis. As shown in Figure 1A, there was significant suppression of IBD in all mice receiving either 500 μg or 50 μg of anti-IL-7Rα M595. The colons from isotype *Hb*-infected mice (positive controls) were irregularly thickened and opaque along the entire length with no formed fecal pellets in the distal colon and showed severe transmural lymphohistiocytic and proliferative colitis (Figure 1B; isotype *Hb*). Anti-IL-7Rα-treated *Hb*-infected mice were normal with well-formed fecal pellets in the distal colon and normal diameter of the proximal colon and were generally considered normal histologically (Figure 1B, anti-IL-7R M595/500 *Hb*) or had some mild focal colitis (anti-IL-7R M595/50 *Hb*). Colons from isotype-broth (negative controls) mice were normal grossly and histologically (Figure 1B, isotype broth). Associated with suppression of IBD, anti-IL-7Rα-treated *Hb*-infected mice had an overall trend of higher percent weight gain compared with *Hb*-infected isotype-treated mice, and the weight gain was similar to uninfected isotype-treated mice (Figure 1C). Mice were analyzed further to determine which immune cells and inflammatory mediators were altered with anti-IL-7Rα M595 treatment.

### Anti-IL-7Rα M595 treatment reduced CD4^+^ and CD8^+^ T cells

**Figure 2:**
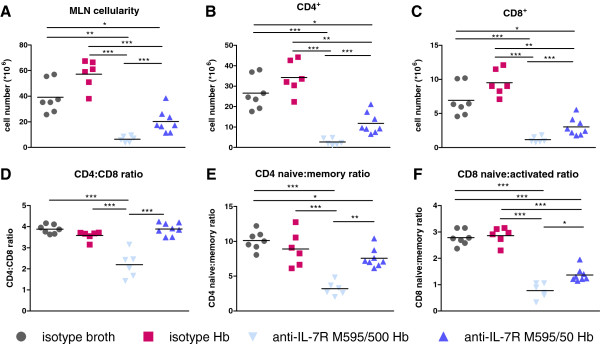
**T-cell numbers were reduced in MLN in *H*. *bilis*-infected *Mdr1a***^−/−^**mice treated with anti-IL-7Rα M595. **Mesenteric lymph nodes (MLN) were harvested from mice and cells were stained with antibodies to identify T-cell subsets by flow cytometry. Total lymphoid cellularity was determined using Advia Hematology Analyzer. (**A**) Total cellularity, (**B**) number of CD4^+^ cells, (**C**) number of CD8^+^ cells, (**D**) CD4^+^ to CD8^+^ T-cell ratio, (**E**) naïve (CD44^-^CD62L^hi^) to activated/memory (CD44^+^CD62L^low^) CD4^+^ T-cell ratio, (**F**) naïve (CD44^-^) to activated/memory (CD44^+^) CD8^+^ T-cell ratio. ANOVA followed by Tukey’s post-test or multivariate t method. *p < 0.05, **p < 0.001, ***p <0.0001.

We focused our analysis on T-cell subsets trafficking to the mesenteric lymph node (MLN) as T-cell expansion is associated with the development of colitis in *Hb*-infected *Mdr1a*^−/−^ mice [11]. Flow cytometric analysis (FACS) revealed that both doses of anti-IL-7Rα M595 significantly prevented the expansion of total MLN cellularity and total CD4^+^ and CD8^+^ T cells compared to both the isotype-broth and isotype-*Hb*-infected mice. Treatment with both anti-IL-7Rα antibody doses further reduced MLN, CD4^+^, and CD8^+^ cellularity below that of *Hb*-infected mice treated with isotype control. However, there were significantly more cells in the anti-IL-7Rα 50 μg-treatment group compared to the 500 μg-treatment group (Figure 2A-C). The higher dose of anti-IL-7Rα M595 resulted in a larger decrease in CD4^+^ T-cell numbers (9.8-fold decrease vs. isotype-broth group) than in CD8^+^ T-cell numbers (4.5-fold decrease vs. isotype-broth group), suggesting total CD4^+^ T cells had a preferential requirement for IL-7R signaling compared to total CD8^+^ T cells. The lower dose of anti-IL-7Rα M595 preserved the ratio of CD4^+^ to CD8^+^ T cells similar to control groups (Figure 2D). We observed similar results for naïve to activated/memory CD4^+^ (Figure 2E) and CD8^+^ (Figure 2F) T-cell ratios with both doses of anti-IL-7Rα M595 treatment. The lower dose of anti-IL-7Rα M595 preserved the ratio of CD4^+^ naïve to memory T cells similar to control groups. There was a greater decrease in absolute numbers of naïve cells versus activated/memory cells (Additional file 1: Figure S1) with anti-IL-7Rα antibody treatment, suggesting that naïve T cells were more sensitive to reduced IL-7R signaling than activated/memory T cells. Our results indicate that a relatively low dose of anti-IL-7Rα M595 can protect mice from *Hb*-induced colitis without inducing lymphopenia to the extent seen with the high dose of anti-IL-7Rα M595. Associated with the alterations in T-cell numbers, we observed a reduction in *Hb*-specific IgG2a antibody titers with anti-IL-7Rα M595 treatment (Additional file 2: Figure S2), suggesting dysregulation of T-dependent-B-cell responses as well.

### Blocking IL-7Rα reduced mediators of colonic inflammation and revealed a role for innate immunity

**Figure 3:**
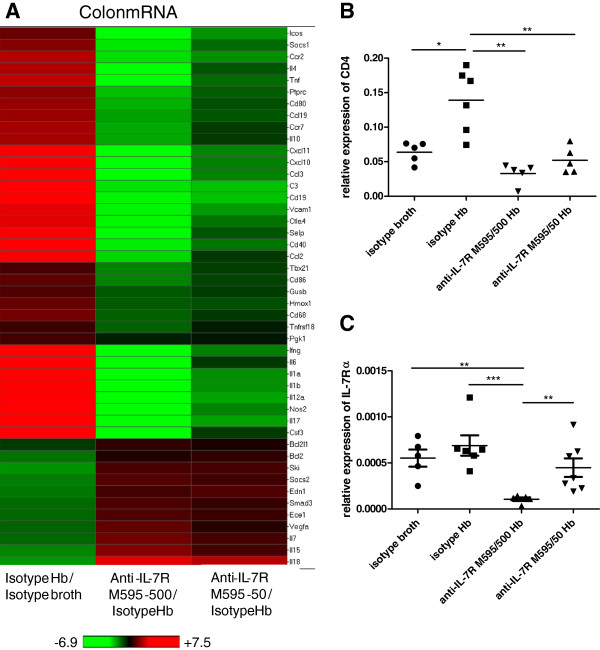
**Messenger RNA expression in *H*. *bilis***-**infected *Mdr1a***^−/−^**mice were altered with anti-IL-7Rα M595 treatment. **Heat maps show hierarchical clustering of expression of colon mRNA (**A**) coding fold changes of the indicated comparisons. (**A**) RNA was extracted from 5 mm of proximal colon from a subset of mice (for isotype *Hb* group, two mice with lower scores and four with higher scores were analyzed). RNA was converted to cDNA and then analyzed using TaqMan® Mouse Immune Array. (**B**) Expression of proximal colon CD4 mRNA (relative to HPRT) from the immune array, and (**C**) proximal colon IL-7Rα mRNA (relative to GAPDH) from single-gene RT-PCR, is shown for individual mice in each treatment group. For (**A**), genes were filtered to show only those that were significantly (p < 0.05) altered between isotype *Hb* and isotype-broth-treatment groups. Red indicates an increase in expression and green indicates a decrease in expression. For (**B**) and (**C**), ANOVA followed by multivariate t method. *p < 0.05, **p < 0.001, ***p < 0.0001.

To investigate how anti-IL-7Rα antibody treatment reduced and/or prevented inflammation in *Hb*-infected *Mdr1a*^-/-^ mice, we analyzed messenger (m) RNA and protein expression in colon tissues. Proximal colon mRNA expression is depicted in a heat map (Figure 3A) comparing expression changes occurring with infection (mean fold change of isotype *Hb* divided by mean fold change of isotype broth) and expression changes when *Hb*-infected mice were treated with anti-IL-7Rα M595 antibody (mean fold change of anti-IL-7R M595 divided by mean fold change of isotype *Hb*). Many cytokines, chemokines, and cell-surface markers associated with inflammatory responses were increased with *Hb* infection and were correspondingly decreased with anti-IL-7Rα M595 treatment (Figure 3A). Although both doses of anti-IL-7Rα M595 clearly prevented the inflammatory response to *Hb* infection, the 500 μg dose had an overall greater effect on regulating gene expression than the 50 μg dose. Specifically, inflammatory cytokines IFN-γ, IL-6, IL-1α, IL-1β, IL-12p35, and IL-17, as well as chemokines strongly induced by IFNγ, such as activated T-cell recruiter, CXCL11 and a more general inflammatory-cell recruiter, CXCL10, followed this pattern. Costimulatory molecules (CD80, CD86, CD40, GITR, and ICOS), adhesion molecules (VCAM-1 and P-selectin), and signaling molecules involved in cytokine regulation (Tbx21 and Socs1) were also increased with *Hb* infection and decreased with anti-IL-7Rα M595 treatment in a dose-dependent manner. Genes involved in apoptosis inhibition (Bcl2, Vegfa) and cytokine modulation (Socs2, Ski, Smad3) were downregulated with *Hb* infection and increased with anti-IL-7Rα M595 treatment. These data also indicate that colonic IL-7 and IL-15 gene expression was downregulated with *Hb* infection, and upregulated with anti-IL-7Rα M595 treatment. The decrease in these two cytokines with infection and increase with anti-IL-7Rα treatment may be due to homeostatic regulation.

RNA expression of immune-cell markers was also altered with anti-IL-7Rα treatment. Similar to changes of CD4^+^ T cell numbers in MLN, colonic CD4 mRNA expression was increased in *Hb*-infected mice and was decreased in *Hb*-infected mice treated with both doses of anti-IL-7Rα M595 (Figure 3B). The effect was specific to CD4 expression (presumably on CD4^+^ T cells) as there were no significant differences in CD3ε or CD8α mRNA expression in colonic tissue (data not shown). Although not significantly increased with *Hb* infection, colonic IL-7Rα mRNA expression was reduced following anti-IL-7Rα M595 treatment. The lower dose of anti-IL-7Rα M595 was not associated with a significant decrease in IL-7Rα mRNA expression compared to either control, whereas the high dose of anti-IL-7Rα M595 had 7-fold less IL-7Rα expression compared to isotype-broth and isotype-*Hb* groups (Figure 3C). Thus, not only did anti-IL-7Rα treatment reduce cellularity in draining lymph nodes, but blocking IL-7Rα reduced immune-cell markers and inflammatory mediators in the colon as well.

*Mdr1a*^−/−^ mice were chosen to test the effects of anti-IL-7Rα in colitis because they are a T- and B-cell sufficient strain. Although many of the changes in gene expression in the colon were related to T cells, several of the regulated genes are involved in innate immunity. For example, genes involved in trafficking and activation of macrophages, natural killer (NK) cells, and dendritic cells (DC) (CCR2, CCL19/CCR7, CCL3, CCL2, Csf3), promotion of cellular responses (CD68, TNFα, IL-10, CCL3, GITR, IL-6, IL-1α, IL-1β, IL-12p35), and innate responses to bacterial infection (C3, CD40, Gusb, Nos2) were upregulated with *Hb* infection and downregulated with anti-IL-7Rα M595 treatment in a dose-dependent manner (Figure 3A).

**Figure 4:**
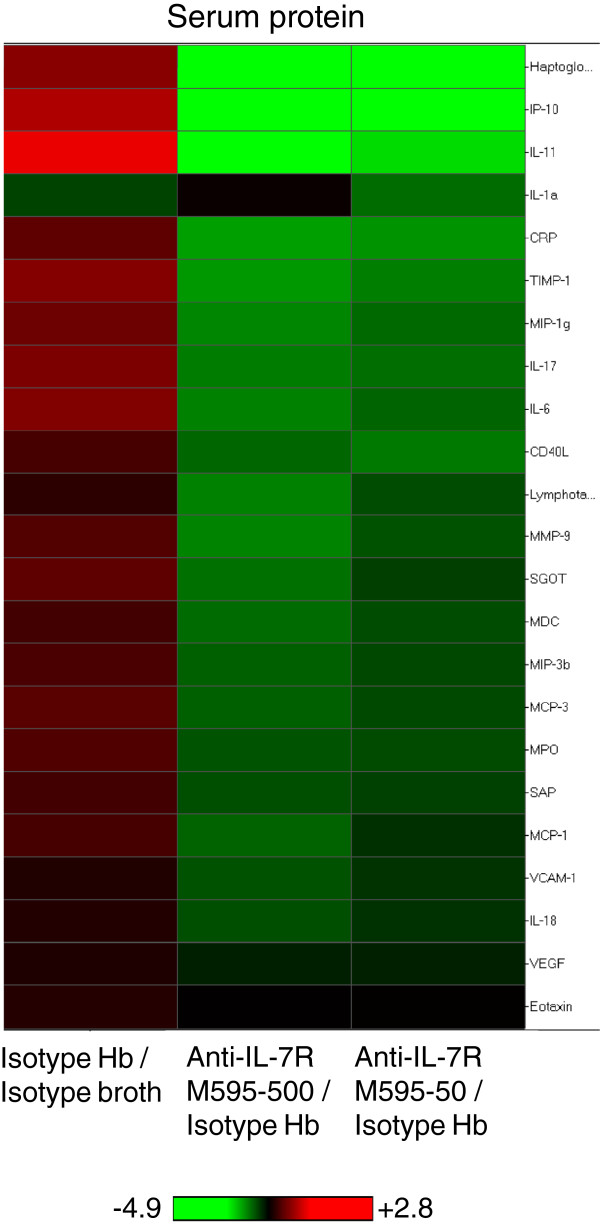
**Serum proteins in *H*. *bilis***-**infected *Mdr1a***^−/−^**mice were altered with anti-IL-7Rα M595 treatment.** Heat maps show hierarchical clustering of expression of serum proteins coding fold changes of the indicated comparisons. Serum was collected at necropsy from the majority of mice and protein expression determined using the RodentMAP version 2.0 antigen panel. Proteins were filtered to show only those that were significantly (p < 0.05) altered between isotype *Hb* and isotype-broth-treatment groups. Red indicates an increase in expression and green indicates a decrease in expression.

**Figure 5:**
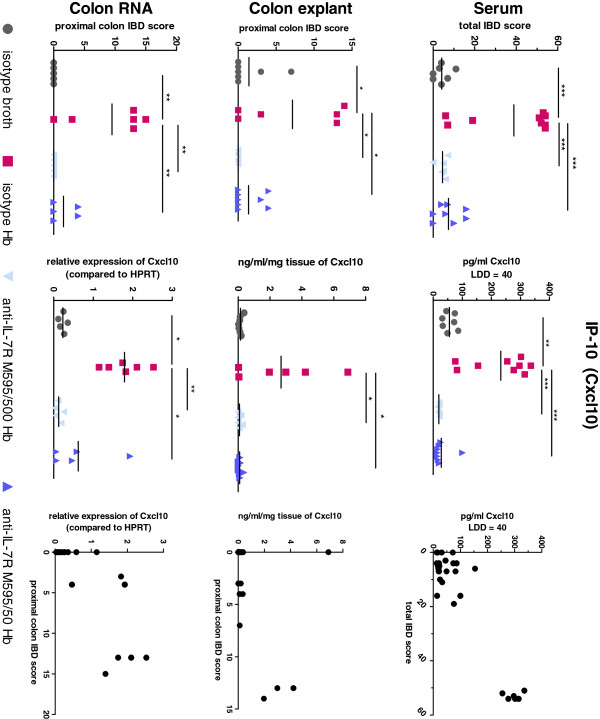
**In *H*. *bilis*-infected *Mdr1a***^−/−^**mice, changes in protein and gene expression of Cxcl10 (IP-10) correlated with histopathology scores.** IBD scores are shown in the left column for the subsets of mice used in each analysis for expression of C-X-C motif chemokine 10 (CXCL10, also known as Interferon gamma-induced protein 10 (IP-10)). The middle column shows serum and colon protein concentrations and colon mRNA levels for the mice in the left column. The graphs in the right column show the comparisons between protein/mRNA and IBD scores (total IBD scores for serum and proximal colon IBD scores for colon explant and mRNA analysis). Mean values for each group are shown for the left and middle columns (horizontal line) as well as statistical significance between groups (analyzed as in Figure 1; *p < 0.05, **p < 0.001, ***p <0.0001). For serum analysis, a least detectable dose was determined for each analyte, and values at or below the limit of detection were assigned a value of one below the least detectable dose for graphing and statistical analysis.

To correlate gene expression changes in colonic tissue with changes in secreted proteins, we analyzed serum samples and proximal colon explant cultures by multi-analyte profiling. Serum protein for IL-17, IL-6, CXCL10 (IP-10), and CCL2 (MCP1) had similar expression-pattern changes to those seen in the gene array, showing increases with *Hb* infection and decreases with anti-IL-7Rα M595 treatment (Figure 4, Additional file 3: Table S1). Additional analytes detected in the serum (but not represented in the gene array panel) also revealed that blocking IL-7Rα regulated innate immune responses. These included MIP-1γ (CCL9), MDC (CCL22), MIP-3β (CCL19), and MPO. Analysis of local production of proteins in colon explants showed expression of CCL3, CXCL10, IFN-γ, IL-17, IL-1α and IL-1β all had similar expression patterns compared to gene expression in adjacent tissue (increased expression with *Hb* infection and a dose-dependent decreased expression with anti-IL-7Rα M595 treatment) (Additional file 3: Table S1). As an example, correlations between histopathology scores, serum and colon protein, and colon mRNA are shown for CXCL10 (IP-10) in Figure 5. Taken together, these results indicate that an innate-immune signature occurred both locally and systemically in *Hb*-infected mice and suggested that an anti-IL-7Rα antibody could regulate adaptive- as well as innate-immune responses in colitis.

### Blocking IL-7Rα ameliorated colitis in the absence of T and B cells

**Figure 6:**
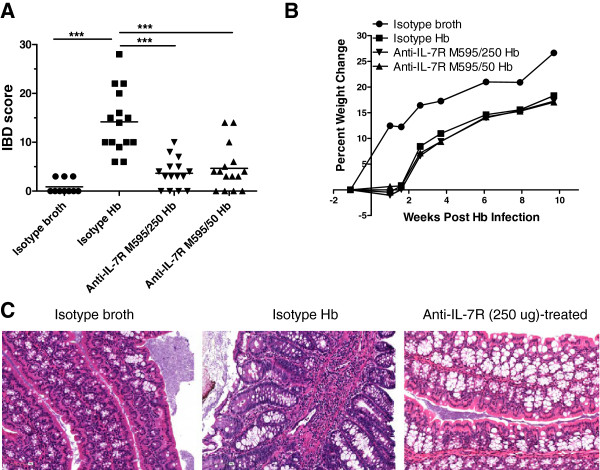
**Treatment with anti-IL-7Rα repressed IBD in T and B-cell deficient *Rag2***^−/−^**mice.** Seven to eleven-week-old *Rag2*^−/−^ mice were treated IP twice weekly with 250 μg or 50 μg of anti-IL-7Rα M595, or 250 μg of isotype control antibody one week prior to oral gavage with *Hb* (1×10^6^ CFU administered twice, on 2 consecutive days) or broth and then treated twice weekly throughout the duration of the study for 11 weeks post-infection. (**A**) Histopathological IBD scores from colon. Cecum and colon IBD scores were based on severity of mucosal epithelial changes, degree of inflammation and extent of lesions [11]. (**B**) Percent change in weight is shown for mice 1 week prior to infection through 10 weeks post infection. (**C**) Representative H&E-stained sections of proximal colon from uninfected control (isotype broth), infected control (isotype *Hb*) and anti-IL-7Rα M595 treated (250 μg) *Hb*-infected mice. Original magnification, 20X. ANOVA followed by Tukey's post-test on square root transformed data (a). *p < 0.05, **p < 0.001, ***p <0.0001.

Given our results successfully treating *Hb*-induced colitis in *Mdr1a*^−/−^ mice by inhibiting IL-7R signaling, we determined whether non-lymphoid cells also respond to anti-IL-7Rα treatment as was suggested in the mRNA and protein analysis. Therefore, we used T- and B-cell deficient *Rag2*^−/−^ mice to determine the effects of blocking IL-7Rα on non-lymphoid cells in *Hb*-induced colitis. We tested anti-IL-7Rα M595 at two doses (250 and 50 μg) in *Hb*-infected-*Rag2*^−/−^ mice. Both doses suppressed inflammation in *Hb*-infected-*Rag2*^−/−^ mice, and they were not statistically different from one another (Figure 6A). Percent weight change was examined throughout the duration of the study. All groups of *Hb*-infected mice had a slight amount of weight loss the first week after infection which did not occur in uninfected mice. Starting at 2 weeks after infection, all groups of *Hb*-infected mice started to gain weight which continued for the duration of the study and were not statistically different than uninfected mice. No differences in percent weight change were seen between *Hb*-infected mice and anti-IL-7Rα-treated *Hb*-infected mice (Figure 6B). Representative H&E-stained sections of proximal colon from uninfected control mice (isotype broth), and *Hb*-infected mice treated with isotype control or anti-IL-7Rα M595 are shown in Figure 6C. *Hb*-induced disease in *Rag2*^−/−^ mice was less severe than that in *Mdr1a*^−/−^ mice and was characterized by moderate hyperplastic and lymphohistiocytic colitis with elongation of colonic glands and expansion of lamina propria and submucosa with inflammatory cells (isotype *Hb*). In contrast, the colons from anti-IL-7Rα treated *Hb*-infected mice had very mild lesions.

### Multiple cell subsets were altered with IL-7Rα blockade in *Rag2*^−/−^ mice

**Figure 7:**
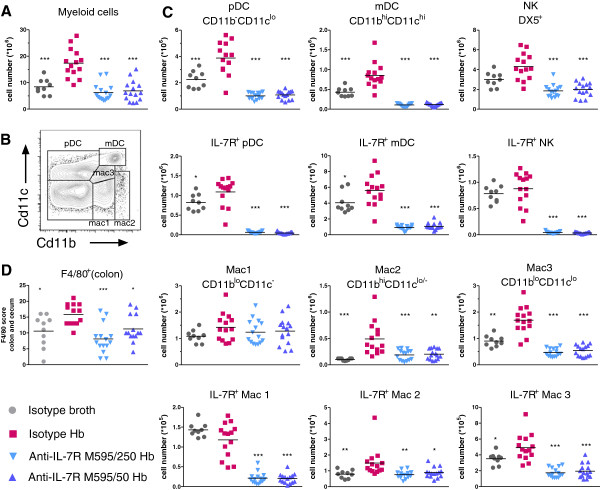
**Splenic macrophages, DCs, and NK cells, and colon F4/80**^**+**^**cells were reduced in *H*. *bilis*-infected *Rag2***^−/−^**mice treated with anti-IL-7Rα M595. **Spleens were harvested from mice and cells were stained with antibodies to identify myeloid-cell subsets by flow cytometry. (**A**) Absolute numbers of splenic myeloid cells were determined using Advia Hematology Analyzer. (**B**) Flow cytometry gating strategy for splenic DC- and macrophage-cell populations using CD11c and CD11b staining. (**C**) Absolute numbers of splenic pDC, mDC, NK cells (gated on DX5^+^ cells), and macrophages, along with corresponding absolute numbers of IL-7R^+^ pDC, mDC, NK cells, and macrophages, were derived from flow cytometry percentages and Advia myeloid-cell counts. (**D**) Sections of colon and cecum were immunostained with F4/80. The F4/80 score is a ratio of the number of F4/80^+^ cells to total section area. ANOVA followed by Dunnett’s or non-parametric *t*-test for all groups vs. isotype *Hb*. *p < 0.05, **p < 0.001, ***p <0.0001.

Given that disruption of IL-7R signaling causes T cells to decline by blocking survival and proliferation signals, it was possible that non-lymphoid cell numbers in the *Rag2*^−/−^ mice could also be affected by IL-7Rα blockade. To determine which cell types were involved in colitic disease in *Hb*-infected *Rag2*^−/−^ mice and whether the anti-IL-7Rα antibodies had any effects on particular cell types, we used FACS to analyze splenic macrophages, DCs, and NK cells. Total myeloid cell numbers were significantly increased with *Hb* infection and significantly decreased with both doses of anti-IL-7Rα M595 (Figure 7A). Separation of splenocytes based on cell surface expression of CD11c and CD11b indicated 5 populations of DCs and macrophages: Plasmacytoid DCs (pDC; CD11b^-^CD11c^lo^), myeloid DCs (mDCs; CD11b^hi^CD11c^hi^), macrophage 1 (Mac1; CD11b^lo^CD11c^-^), macrophage 2 (Mac2: CD11b^hi^ CD11c^lo/-^) and macrophage 3 (Mac3; CD11b^lo^CD11c^lo^) (Figure 7B). We enumerated NK cells using surface expression of DX5. The expression of IL-7Rα on each subtype was determined, and numbers of total or IL-7Rα^+^ pDC, mDC, NK, Mac2, and Mac3 cells were increased with *Hb* infection (Figure 7C). Mac1 cell numbers were not altered significantly with disease. Treatment of mice with anti-IL-7Rα antibodies either maintained cell numbers similar to uninfected mice or lowered cell numbers further from uninfected control levels (Figure 7C). Some IL-7Rα^+^ cell subsets appeared to be more sensitive to anti-IL-7Rα M595 antibody treatment than others. For example, the decrease in cell numbers compared to the uninfected isotype control group was greater for IL-7Rα^+^ pDC than for total pDC (13- vs. 2.2-, and 18.6- vs. 2.1-fold decrease in M595/250 and M595/50 groups, respectively). This was also true for NK cells where anti-IL-7Rα M595 treatment groups had 17- and 22-fold fewer numbers (M595/250 and M595/50 groups, respectively) of IL-7Rα^+^ NK cells compared to uninfected isotype controls. This was in contrast to total NK cells in which anti-IL-7Rα-M595-treated groups had similar NK-cell numbers to uninfected control mice. Unlike the results in *Mdr1a*^−/−^ mice, there were no significant differences between anti-IL-7Rα M595 doses on IBD scores or cell numbers in these studies. In addition, we used F4/80 immunohistochemistry to identify macrophage/DCs in proximal colon (Figure 7D). Consistent with findings in spleen for macrophages and DCs, F4/80^+^ cells in the colon were increased with disease and treatment with anti-IL-7Rα M595 preserved F4/80^+^ cell numbers similar to uninfected control mice. These observations strongly suggest that innate immune cells, including those expressing IL-7Rα, are involved in *Hb*-induced colitis, and that anti-IL-7Rα can limit their expansion and protect from development of colitis.

### Anti-IL-7Rα treatment of *Hb*-infected *Rag2*^−/−^ mice reduced mediators of colonic inflammation

**Figure 8:**
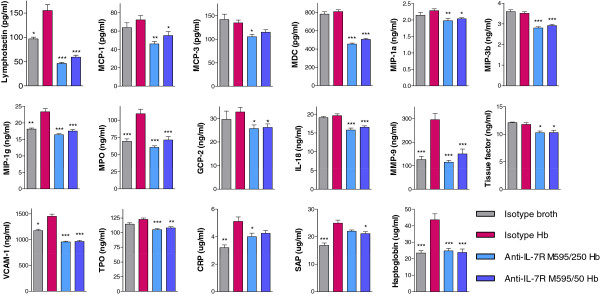
**Circulating mediators involved in colon inflammation and damage were reduced in *H*. *bilis***-**infected *Rag2***^−/−^**mice treated with anti-IL-7Rα M595. **Serum was collected at necropsy and protein expression determined using the RodentMAP version 2.0 antigen panel. ANOVA followed by Dunnett’s post-test for all groups vs. isotype *Hb*. *p < 0.05, **p < 0.001, ***p <0.0001.

To assess the activity of innate cells, we measured serum proteins by multi-analyte profiling. In the absence of T and B cells, there were fewer analytes altered with infection or with anti-IL-7Rα M595 treatment compared with the number of changes seen in *Hb*-infected *Mdr1a*^−/−^ mice. However, the majority of analytes altered in these mice were chemotactic proteins (Figure 8). Circulating concentrations of lymphotactin, MIP-1γ, MPO, MMP9, as well as VCAM-1, CRP, SAP, and haptoglobin were altered with *Hb*-infection and reduced in *Hb*-infected mice treated with anti-IL-7Rα M595. Some analytes were not significantly increased with infection but were significantly reduced by treatment with the anti-IL-7Rα M595: MCP-1, MCP-3, MDC, MIP-1α, MIP3β, GCP-2, IL-18, Tissue Factor, and TPO. Thus, in line with the reduction in myeloid cells, innate-immune mediators were also reduced with IL-7Rα blockade and strongly indicate that anti-IL-7Rα treatment controls colitis by multiple mechanisms.

## Discussion

We used two mouse models of bacterial-induced colitis to study the effects of blocking IL-7Rα on IBD: a T- and B-cell-sufficient strain (*Mdr1a*^−/−^) and a T- and B-cell-deficient strain (*Rag2*^−/−^). These models were used to circumvent the confounding variable of IL-7-dependent expansion of T cells as occurs in colitis models that rely on adoptive transfer of T cells into a lymphopenic environment. Our results show that IL-7Rα blockade can ameliorate bacterial-induced colitis, and that this effect involves not only T cells but also innate immune cells such as macrophages, DC, and NK cells. Successful treatment of colitis with an anti-IL-7Rα antibody was associated with decreases in T-cell and non-T-cell populations, as well as a reduction of inflammatory cytokines and chemokines. Our results confirm that IL-7Rα^+^ T-cell-mediated activities are likely key players in IBD. However, our results differ from some reports in that we demonstrate that IL-7R-mediated activity on non-T and non-B cells also contributes to experimental colitis.

In TCRα^−/−^ mice and T-cell-transfer-induced colitis, high expression of IL-7Rα on T cells is associated with colitis [9,10,15], and IL-7-producing bone marrow cells may harbor colitogenic memory CD4^+^ T cells [16]. The use of a toxin-conjugated anti-IL-7Rα antibody (A7R34) by Yamazaki and colleagues suggests that selective elimination of IL-7Rα^high^ CD4^+^ LPL is required to treat colitis [10]. In our studies with *Mdr1a*^−/−^ mice, we noted that all T-cell subsets in draining MLN were decreased with anti-IL-7Rα M595 treatment, indicating that decreasing total T cells (which likely included CD4^+^IL-7Rα^high^ cells) was in large part responsible for the lack of colitis seen in anti-IL-7Rα M595 antibody-treated mice. We did not detect increased colonic expression of IL-7Rα mRNA in *Hb*-infected *Mdr1a*^−/−^ mice. The apparent discrepancy between our results of reducing total T cells vs. that of other’s work specifically eliminating IL-7Rα^high^ CD4^+^ T cells may be due to the different model systems. In our models, *Hb* is driving chronic inflammation by both adaptive and innate cells, whereas the adoptive transfer models are skewed towards promoting IL-7Rα^high^ T-cell expression and expansion. For example, in the 500 μg M595-treated *Hb*-infected *Mdr1a*^−/−^ mice, we saw a greater decrease in total CD4^+^ vs. CD8^+^ T cells, as well as in naïve vs. activated/memory CD4^+^ and CD8^+^ T cells. Naïve T cells express high levels of IL-7Rα, whereas activated T cells down regulate IL-7Rα [17]. Our FACS analysis did not distinguish between activated effector cells and memory cells, but this total population was less affected by IL-7Rα blockade than naïve T cells, perhaps due to lower expression of IL-7Rα. Reducing the pool of naïve T-cells may help control colitis by removing cells that would become activated or develop into memory cells following exposure to *Hb*. Regardless of whether anti-IL-7Rα efficacy is due to specifically decreasing IL-7Rα^high^ or IL-7Rα^+^ T cells in general, the combined effect of reducing both naïve and activated/memory T cells was associated with reduced colitis in *Mdr1a*^−/−^ mice.

In *Hb*-infected *Mdr1a*^−/−^ mice, the lower dose of anti-IL-7Rα M595 (50 μg) was as effective in preventing colitis as the higher dose (500 μg), despite the lower dose having less of an effect on reducing T-cell numbers (in MLN). In fact, the lower dose preserved ratios of CD4^+^ to CD8^+^ and naïve to memory CD4^+^ cells in MLN similar to control levels. In the colon however, the reduction in CD4 mRNA expression was the same with both doses of M595. The lower dose of M595 was also effective in reducing inflammatory mediators as measured in colon and serum, indicating that a relatively low dose of M595 can also inhibit the function of T cells (and likely non-T cells). The mechanism by which M595 antibody decreases cellularity is likely due more to population decay by inhibiting survival signals [18,19] rather than direct depletion of target cells. However, antibody-dependent cell-mediated cytotoxicity (ADCC) measurements of M595 showed it to be weakly lytic (Amgen Inc.; data not shown). It would be interesting to perform these colitis studies with a re-engineered anti-IL-7Rα antibody that does not have the confounding variable of ADCC. Further dose–response studies would be needed, but these results suggest there may be a therapeutic window for anti-IL-7Rα in inflammatory disease settings whereby T-cell function would be controlled without significant lymphopenia.

Given the significant alterations of chemokines and cytokines associated with innate immune cells in the serum and colon in the *Mdr1a*^−/−^ model, we explored anti-IL-7Rα treatment in a *Rag2*^−/−^ model of colitis. Amelioration of disease with anti-IL-7Rα treatment in the *Rag2*^−/−^ colitis model suggested that IL-7Rα expressing non-T cells, including macrophages, DCs, and NK cells are important in bacterial-induced disease. In fact, there were moderate (but significant) increases in surface expression of IL-7Rα on splenic macrophage and DC populations in *Hb*-infected-*Rag2*^−/−^ mice. Multiple myeloid cell populations, along with inflammatory cytokines and chemokines associated with their function, were decreased with anti-IL-7Rα antibody treatment in *Hb*-infected *Rag2*^−/−^ mice. Our findings differ from the conclusions reported by Shinohara and colleagues in which they used an adoptive transfer model of colitis to show that expression of IL-7Rα on CD4^+^ T cells, but not on other cells (NK cells, granulocytes, macrophages, and DC), was essential for development of colitis [20]. However, the adoptive transfer model of colitis is dependent on IL-7R-mediated expansion of T cells for induction of colitis, in contrast to our models wherein bacterial antigens drive inflammation and colitis involving resident host T cells (when present) and other innate immune cells. It is possible that IL-7R expression by cells other than CD4^+^ T cells has a modest effect in their model as they noted a trend of increased colitis scores in *IL7R*^−/−^ x *Rag2*^−/−^ recipients of wildtype naïve T cells compared to *Rag2*^−/−^ -only recipients, although these differences were not significant [20].

Our studies using *Mdr1a*^−/−^ and *Rag2*^−/−^ mice strongly suggest that cells other than CD4^+^ T cells are important in bacterial-induced colitis. DCs along with other APCs, are likely involved in the development of colitis [21]. In *Hb*-infected *Rag2*^−/−^ mice, we found that both pDCs and mDCs were significantly increased with colitis and dramatically decreased with anti-IL-7Rα treatment, especially IL-7Rα^+^ pDCs. In *Mdr1a*^−/−^ mice, DC-derived cytokines and DC-activation markers CD80 and CD86 were increased with disease and significantly decreased with anti-IL-7Rα antibody treatment. These results are highly consistent with other studies in T-cell sufficient mice, where IL-7 production from DCs and/or IL-7R signaling in DCs may regulate the proliferation and activation status of CD4^+^ T cells [22] involved in colitis.

Our results showed that NK cells may be associated with *Hb*-induced colitis. We found that lymphotactin, produced by and chemotactic for NK cells, was increased with disease and decreased following anti-IL-7Rα M595 treatment in both *Rag2*^−/−^ and *Mdr1a*^−/−^ mice infected with *Hb*. A regulatory role for NK cells was demonstrated in one adoptive transfer model of IBD [23], whereas another study showed NK cells neither suppressed nor exacerbated adoptive transfer-induced IBD [24]. Our findings using *Rag2*^−/−^ mice infected with *Hb* indicated that total NK and IL-7Rα^+^ NK cells were associated with promoting IBD rather than suppression. Further studies using various IBD models are needed to clarify the function of NK cells in experimental colitis.

IL-7 promotes inflammation in part via activation of monocytes and macrophages [25] and induces proinflammatory cytokines and chemokines. In our two models, blockade of IL-7Rα decreased colonic F4/80^+^ cells and CD68 mRNA in *Hb*-infected *Mdr1a*^−/−^ mice, decreased subpopulations of macrophages in *Hb*-infected *Rag2*^−/−^ mice, and reduced monocyte-derived chemokines in both *Hb*-infected *Mdr1a*^−/−^ and *Rag2*^−/−^ mice. Shinohara and colleagues report that only IL-7R^+^CD4^+^ T cells (and not NK cells, granulocytes, DCs, or macrophages) contribute to colitis in their adoptive transfer model [20], whereas von Freeden-Jeffry and colleagues reported that in the absence of T and B cells, IL-7 dramatically increases F4/80^+^ cell infiltration into the intestinal mucosa upon *H*. *hepaticus* infection resulting in chronic intestinal inflammation [8]. The main difference between these studies is the use of adoptive transfer of T cells into a lymphopenic environment vs. use of a bacterium to trigger IBD. Whereas T cells are important for IBD pathogenesis, our conclusions that non-T cells are also important in intestinal inflammation is aligned with von Freeden-Jeffry’s results. Our results using pharmacological blockade of IL-7Rα in T-cell sufficient and T- and B-cell-deficient mice clearly establish the contributions of both T cells and non-T cells such as macrophages, DCs and NK cells in IBD pathogenesis.

## Conclusions

In summary, our studies are consistent with other models of gastrointestinal inflammation in which anti-IL-7Rα antibody therapy ameliorated IBD [10,15,26]. However, we further show using a bacterial trigger of colitis in a T-cell competent model and a non-T non-B cell model, that blocking IL-7Rα can decrease inflammatory responses via its effect on multiple immune cells that include not only T cells, but also B cells, macrophages, DC, and NK cells. Importantly, complete depletion of T-cells (or myeloid cells) was not required to successfully treat colitis. Our studies demonstrate an important function of IL-7R-driven immunity in experimental colitis and indicate that the therapeutic efficacy of IL-7Rα blockade involves affecting both adaptive and innate immunity.

## Competing interests

CRW, LN, CLS, JM, and GC were employed by Amgen, Inc. at the time the studies were completed. This work was sponsored by Amgen, Inc. LMP received funding from Amgen, Inc. for portions of the studies conducted at the University of Washington. AS, TB, PT, SP, and BMI declare that they have no competing interests.

## Authors’ contributions

CRW and AS designed, conducted, analyzed and interpreted data, prepared figures, drafted and revised the manuscript. JM, TB, LMP designed, analyzed and interpreted data, and edited the manuscript. PT performed histopathology analysis and interpretation, and edited the manuscript. LN designed, performed, and analyzed flow cytometry data. GC performed statistical analysis and interpretation and designed the heat maps. SP performed the in vivo portions of the studies and tissue processing. CLS analyzed serum antibody concentrations. BMI contributed to data interpretation and editing of the manuscript. All authors have read and approved this manuscript.

## Authors’ information

Joint first authors: Cynthia R Willis and Audrey Seamons.

## Supplementary Material

Additional file 1 **Figure S1. **Naïve and activated/memory T cells were reduced in MLN in *H*. *bilis*-infected *Mdr1a*^−/−^ mice treated with anti-IL-7Rα M595. MLN were harvested from mice shown in Figure 1a and cells were stained with antibodies to identify T-cell subsets by flow cytometry. Cell numbers of MLN (A) naïve (CD44^-^CD62Lhi), (B) activated/memory (CD44^+^CD62L^low^) CD4^+^ T cells, (C) naïve (CD44^-^), and (D) activated/memory (CD44^+^) CD8^+^ T cells are shown. Significant differences are shown (ANOVA followed by Tukey’s post-test or multivariate t method. *p < 0.05, **p < 0.001, ***p <*0*.*0001*). Click here for file

Additional file 2 **Figure S2. **Circulating concentrations of *H*. *bilis*-specific antibodies were lower in infected *Mdr1a*^−/−^ mice treated with anti-IL-7Rα M595. Serum was collected from mice shown in Figure 1a and analyzed for *Hb*-specific IgG2a by ELISA. Anti-*Hb* absorbance was determined by calculating the average absorbance of each sample minus the average absorbance of the control wells. Data represent the mean ± SEM for each treatment group at each dilution. For statistical calculations, the optical density of each sample was multiplied by the dilution factor, then the values obtained in the 500-, 5000-, and 50,000-fold dilution groups were summed. The two anti-IL-7Rα M595-treated groups were compared to the isotype control group by taking the rank transformation of the data and performing ANOVA with Dunnett’s post-test. *p < *0*.*05*, **p < *0*.*01*. Click here for file

Additional file 3 **Table S1. **Expression of serum and colon explant proteins and colon mRNA that were altered with colitis and with anti-IL-7Rα M595 antibody treatment in *Hb*-infected *Mdr1a*^-/-^ mice. Click here for file

## References

[B1] Di Sabatino A Biancheri P Rovedatti L Macdonald TT Corazza GR New pathogenic paradigms in inflammatory bowel disease Inflamm Bowel Dis 2012 18 368 371 10.1002/ibd.21735 21538717

[B2] Ma A Koka R Burkett P Diverse functions of IL-2, IL-15, and IL-7 in lymphoid homeostasis Annu Rev Immunol 2006 24 657 679 10.1146/annurev.immunol.24.021605.090727 16551262

[B3] Watanabe M Watanabe N Iwao Y Ogata H Kanai T Ueno Y Tsuchiya M Ishii H Aiso S Habu S Hibi T The serum factor from patients with ulcerative colitis that induces T cell proliferation in the mouse thymus is interleukin-7 J Clin Immunol 1997 17 282 292 10.1023/A:1027322631036 9258767

[B4] Kader HA Tchernev VT Satyaraj E Lejnine S Kotler G Kingsmore SF Patel DD Protein microarray analysis of disease activity in pediatric inflammatory bowel disease demonstrates elevated serum PLGF, IL-7, TGF-beta1, and IL-12p40 levels in Crohn's disease and ulcerative colitis patients in remission versus active disease Am J Gastroenterol 2005 100 414 423 10.1111/j.1572-0241.2005.40819.x 15667502 PMC1586185

[B5] Futagami S Hiratsuka T Suzuki K Kusunoki M Wada K Miyake K Ohashi K Shimizu M Takahashi H Gudis K Gammadelta T cells increase with gastric mucosal interleukin (IL)-7, IL-1beta, and Helicobacter pylori urease specific immunoglobulin levels via CCR2 upregulation in Helicobacter pylori gastritis J Gastroenterol Hepatol 2006 21 32 40 10.1111/j.1440-1746.2005.04148.x 16706809

[B6] Anderson CA Boucher G Lees CW Franke A D'Amato M Taylor KD Lee JC Goyette P Imielinski M Latiano A Meta-analysis identifies 29 additional ulcerative colitis risk loci, increasing the number of confirmed associations to 47 Nat Genet 2011 43 246 252 10.1038/ng.764 21297633 PMC3084597

[B7] Watanabe M Ueno Y Yajima T Okamoto S Hayashi T Yamazaki M Iwao Y Ishii H Habu S Uehira M Interleukin 7 transgenic mice develop chronic colitis with decreased interleukin 7 protein accumulation in the colonic mucosa J Exp Med 1998 187 389 402 10.1084/jem.187.3.389 9449719 PMC2212121

[B8] von Freeden-Jeffry U Davidson N Wiler R Fort M Burdach S Murray R IL-7 deficiency prevents development of a non-T cell non-B cell-mediated colitis J Immunol 1998 161 5673 5680 9820548

[B9] Okada E Yamazaki M Tanabe M Takeuchi T Nanno M Oshima S Okamoto R Tsuchiya K Nakamura T Kanai T IL-7 exacerbates chronic colitis with expansion of memory IL-7Rhigh CD4+ mucosal T cells in mice Am J Physiol Gastrointest Liver Physiol 2005 288 G745 G754 10.1152/ajpgi.00276.2004 15550560

[B10] Yamazaki M Yajima T Tanabe M Fukui K Okada E Okamoto R Oshima S Nakamura T Kanai T Uehira M Mucosal T cells expressing high levels of IL-7 receptor are potential targets for treatment of chronic colitis J Immunol 2003 171 1556 1563 12874249 10.4049/jimmunol.171.3.1556

[B11] Maggio-Price L Shows D Waggie K Burich A Zeng W Escobar S Morrissey P Viney JL Helicobacter bilis infection accelerates and H. hepaticus infection delays the development of colitis in multiple drug resistance-deficient (mdr1a−/−) mice Am J Pathol 2002 160 739 751 10.1016/S0002-9440(10)64894-8 11839595 PMC1850632

[B12] Maggio-Price L Bielefeldt-Ohmann H Treuting P Iritani BM Zeng W Nicks A Tsang M Shows D Morrissey P Viney JL Dual infection with Helicobacter bilis and Helicobacter hepaticus in p-glycoprotein-deficient mdr1a−/− mice results in colitis that progresses to dysplasia Am J Pathol 2005 166 1793 1806 10.1016/S0002-9440(10)62489-3 15920164 PMC1602406

[B13] Burich A Hershberg R Waggie K Zeng W Brabb T Westrich G Viney JL Maggio-Price L Helicobacter-induced inflammatory bowel disease in IL-10- and T cell-deficient mice Am J Physiol Gastrointest Liver Physiol 2001 281 G764 G778 11518689 10.1152/ajpgi.2001.281.3.G764

[B14] Brabb T von Dassow P Ordonez N Schnabel B Duke B Goverman J In situ tolerance within the central nervous system as a mechanism for preventing autoimmunity J Exp Med 2000 192 871 880 10993917 10.1084/jem.192.6.871 PMC2193284

[B15] Totsuka T Kanai T Nemoto Y Makita S Okamoto R Tsuchiya K Watanabe M IL-7 Is essential for the development and the persistence of chronic colitis J Immunol 2007 178 4737 4748 17404253 10.4049/jimmunol.178.8.4737

[B16] Nemoto Y Kanai T Makita S Okamoto R Totsuka T Takeda K Watanabe M Bone marrow retaining colitogenic CD4+ T cells may be a pathogenic reservoir for chronic colitis Gastroenterology 2007 132 176 189 10.1053/j.gastro.2006.10.035 17241870

[B17] Fry TJ Mackall CL The many faces of IL-7: from lymphopoiesis to peripheral T cell maintenance J Immunol 2005 174 6571 6576 15905493 10.4049/jimmunol.174.11.6571

[B18] Fry TJ Mackall CL Interleukin-7: from bench to clinic Blood 2002 99 3892 3904 10.1182/blood.V99.11.3892 12010786

[B19] Vivien L Benoist C Mathis D T lymphocytes need IL-7 but not IL-4 or IL-6 to survive in vivo Int Immunol 2001 13 763 768 10.1093/intimm/13.6.763 11369703

[B20] Shinohara T Nemoto Y Kanai T Kameyama K Okamoto R Tsuchiya K Nakamura T Totsuka T Ikuta K Watanabe M Upregulated IL-7 receptor (alpha) expression on colitogenic memory CD4+ T cells may participate in the development and persistence of chronic colitis J Immunol 2011 186 2623 2632 10.4049/jimmunol.1000057 21217010

[B21] Coombes JL Powrie F Dendritic cells in intestinal immune regulation Nat Rev Immunol 2008 8 435 446 10.1038/nri2335 18500229 PMC2674208

[B22] Guimond M Veenstra RG Grindler DJ Zhang H Cui Y Murphy RD Kim SY Na R Hennighausen L Kurtulus S Interleukin 7 signaling in dendritic cells regulates the homeostatic proliferation and niche size of CD4+ T cells Nat Immunol 2009 10 149 157 19136960 10.1038/ni.1695 PMC2713006

[B23] Fort MM Leach MW Rennick DM A role for NK cells as regulators of CD4+ T cells in a transfer model of colitis J Immunol 1998 161 3256 3261 9759840

[B24] Laroux FS Norris HH Houghton J Pavlick KP Bharwani S Merrill DM Fuseler J Chervenak R Grisham MB Regulation of chronic colitis in athymic nu/nu (nude) mice Int Immunol 2004 16 77 89 10.1093/intimm/dxh006 14688063

[B25] van Roon JA Verweij MC Wijk MW Jacobs KM Bijlsma JW Lafeber FP Increased intraarticular interleukin-7 in rheumatoid arthritis patients stimulates cell contact-dependent activation of CD4(+) T cells and macrophages Arthritis Rheum 2005 52 1700 1710 10.1002/art.21045 15934068

[B26] Ohana M Okazaki K Oshima C Andra's D Nishi T Uchida K Uose S Nakase H Matsushima Y Chiba T A critical role for IL-7R signaling in the development of Helicobacter felis-induced gastritis in mice Gastroenterology 2001 121 329 336 10.1053/gast.2001.26289 11487542

