# Supplementary figures and images for: Interleukin-7 receptor blockade suppresses adaptive and innate inflammatory responses in experimental colitis
Source: J Inflamm (Lond). 2012 Oct 12;9:39. doi: 10.1186/1476-9255-9-39 (PMC3551718; doi:10.1186/1476-9255-9-39)

Supplementary Figure 1

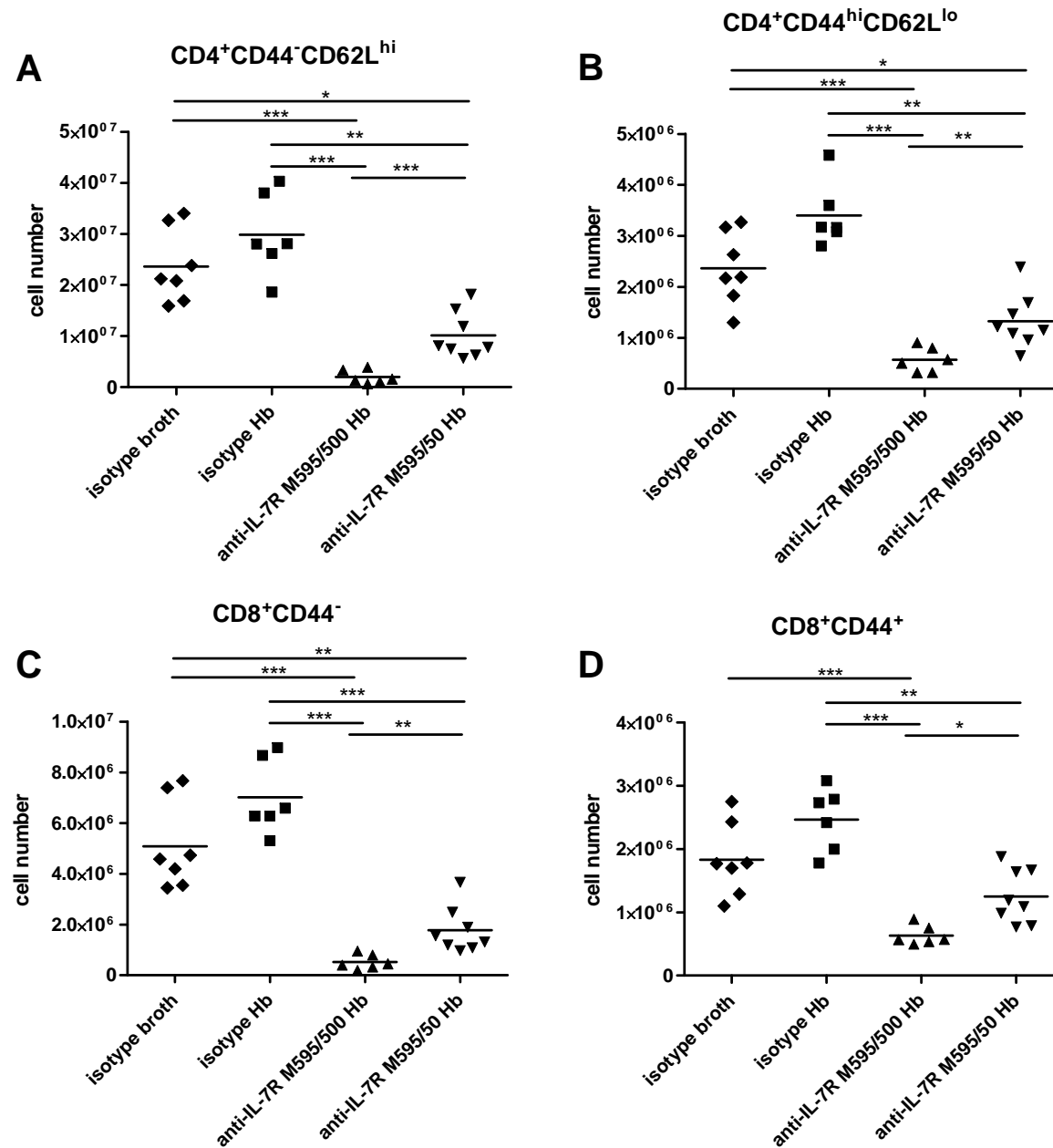

Supplement: Additional file 1 — Figure S1. Naïve and activated/memory T cells were reduced in MLN in H. bilis-infected Mdr1a−/− mice treated with anti-IL-7Rα M595. MLN were harvested from mice shown in Figure 1a and cells were stained with antibodies to identify T-cell subsets by flow cytometry. Cell numbers of MLN (A) naïve (CD44-CD62Lhi), (B) activated/memory (CD44+CD62Llow) CD4+ T cells, (C) naïve (CD44-), and (D) activated/memory (CD44+) CD8+ T cells are shown. Significant differences are shown (ANOVA followed by Tukey’s post-test or multivariate t method. *p < 0.05, **p < 0.001, ***p <0.0001). [file 1476-9255-9-39-S1.pdf]

Supplementary Figure 2

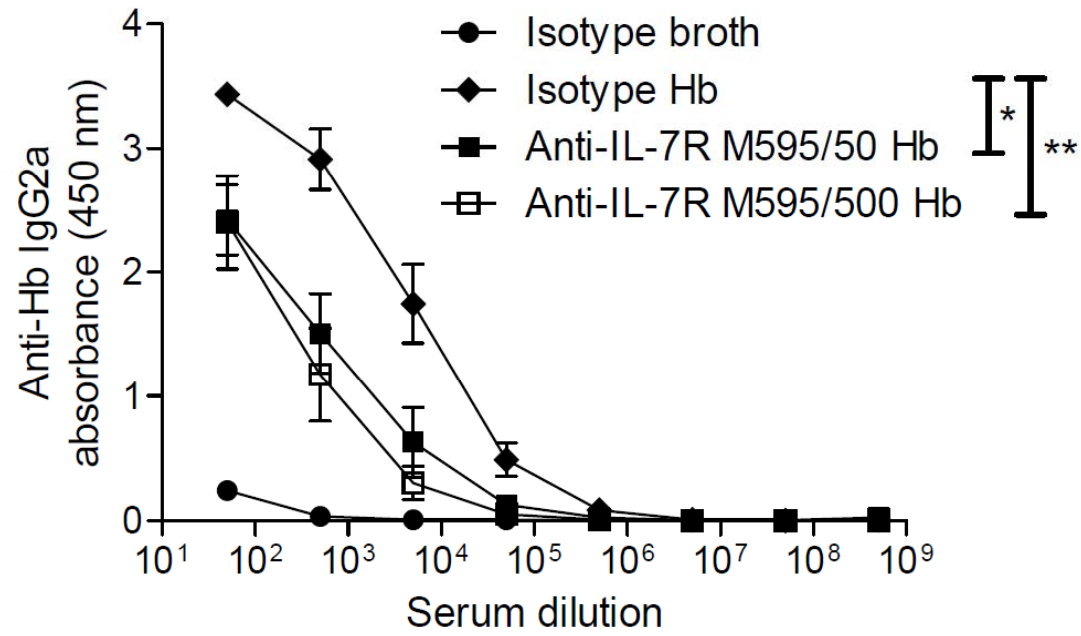

Supplement: Additional file 2 — Figure S2. Circulating concentrations of H. bilis-specific antibodies were lower in infected Mdr1a−/− mice treated with anti-IL-7Rα M595. Serum was collected from mice shown in Figure 1a and analyzed for Hb-specific IgG2a by ELISA. Anti-Hb absorbance was determined by calculating the average absorbance of each sample minus the average absorbance of the control wells. Data represent the mean ± SEM for each treatment group at each dilution. For statistical calculations, the optical density of each sample was multiplied by the dilution factor, then the values obtained in the 500-, 5000-, and 50,000-fold dilution groups were summed. The two anti-IL-7Rα M595-treated groups were compared to the isotype control group by taking the rank transformation of the data and performing ANOVA with Dunnett’s post-test. *p < 0.05, **p < 0.01. [file 1476-9255-9-39-S2.pdf]
