# Supplementary material for: Interleukin-7 receptor blockade suppresses adaptive and innate inflammatory responses in experimental colitis
Source: J Inflamm (Lond). 2012 Oct 12;9:39. doi: 10.1186/1476-9255-9-39 (PMC3551718; doi:10.1186/1476-9255-9-39)
Supplement: Additional file 3 — Table S1. Expression of serum and colon explant proteins and colon mRNA that were altered with colitis and with anti-IL-7Rα M595 antibody treatment in Hb-infected Mdr1a-/- mice. [file 1476-9255-9-39-S3.doc]

| **Additional file 3 Table S1**. Expression of serum and colon explant proteins and colon mRNA that were altered with colitis and with anti-IL-7Rα M595 antibody treatment in *Hb*-infected *Mdr1a^-/-^* mice. | | | | | | | | |
| --- | --- | --- | --- | --- | --- | --- | --- | --- |
| **Category** | **Name** | **Serum** | **Serum correlation w/ disease*,**^†^ | **Colon Explant** | **Colon explant correlation w/ disease*,**^†^ | **Immune array (colon)** | **Immune array correlation w/ disease*,**^†^ | |
| chemokines/ chemokine receptors | Ccl19 (MIP-3β) | **↑1.8 ↓2.2** ↓**1.8** | Yes | nd | nd | **↑3.5 ↓3.7 ↓2.8** | | yes |
|  | Ccl2 (MCP-1) | **↑1.8** **↓2.3**,**↓**1.5 | r = 0.5547  p=0.0015 | same | no | **↑7.8 ↓5.7** **↓**1.6 | | yes |
|  | Ccl3 (MIP-1α) | **↑**1.2 **↓1.5 ↓1.4** | yes | **↑**5.5 **↓**4.9 **↓**5.5 | no | **↑11.4** **↓12.3** **↓2.9** | | yes |
|  | Ccl5 (Rantes) | **↑**1.7 **↓3.1** **↓5.7** | nd | **↑**2.6 **↓**3.7 **↓5.0** | no | **↑**1.4 **↓2.4 ↓1.9** | | no |
|  | Ccl7 (MCP-3) | **↑2.1 ↓2.2 ↓1.8** | yes | nd | nd | nd | | nd |
|  | Ccl9 (Mip-1γ) | **↑2.5 ↓3.0 ↓2.4** | r=0.5167  p=0.0035 | nd | nd | nd | | nd |
|  | Ccl11 (eotaxin) | **↑1.3 ↓**1.0 **↓**1.0 | no | nd | nd | nd | | nd |
|  | Ccl22 (MDC) | **↑1.7 ↓2.4 ↓1.9** | p = 0.0017  r = 0.5486 | nd | nd | nd | | nd |
|  | Ccr2 | nd | nd | nd | nd | **↑4.4 ↓6.2 ↓3.2** | | yes |
|  | Ccr7 | nd | nd | nd | nd | **↑3.8 ↓5.8** **↓**1.6 | | yes |
|  | Cxcl10 (IP-10) | **↑4.2 ↓12.0 ↓8.5** | yes | **↑**18.8 **↓30.3 ↓27.5** | r=0.4067  p=0.0353 | **↑7.8 ↓14.2 ↓2.9** | | yes |
|  | Cxcl11 (I-TAC) | nd | nd | nd | nd | **↑7.5 ↓19.3 ↓3** | | yes |
|  | Cxcl19 (MIG) | nd | nd | **↑16.1 ↓51.0 ↓18.8** | p = 0.0057  r = 0.5172 | nd | | nd |
|  | XCL-1 (Lymphotactin) | **↑1.4 ↓2.9 ↓1.9** | no | nd | nd | nd | | nd |
|  |  |  |  |  |  |  | |  |
| leukocyte phenotypic markers | Cd19 | nd | nd | nd | nd | **↑11.0 ↓6.6 ↓5.0** | | r=0.6591  p= 0.0012 |
|  | Cd4 | nd | nd | nd | nd | **↑2.2 ↓4.2 ↓2.7** | | yes |
|  | Cd68 | nd | nd | nd | nd | **↑2.5 ↓2.2 ↓1.6** | | yes |
|  | H2-Eα | nd | nd | nd | nd | **↑**1.6 **↓1.8** **↓**1.7 | | no |
|  | IL-2Rα (CD25) | nd | nd | nd | nd | **↑**2.3 **↓7.9** **↓**1.7 | | r=0.6769  p= 0.0008 |
|  | Tnfrsf18 (Gitr) | nd | nd | nd | nd | **↑1.6 ↓2.2** **↓**1.3 | | yes |
|  | IL-7R* | nd | nd | nd | nd | **↑**1.2 **↓6.5** **↓**1.5 | | no |
|  |  |  |  |  |  |  | |  |
| co-stimulatory molecules | Cd40 | Same | no | nd | nd | **↑9.2 ↓7.0 ↓2.6** | | yes |
|  | Cd40 ligand | **↑1.8 ↓2.3 ↓2.7** | yes | nd | nd | **↑**3.1 **↓15.0 ↓5.5** | | yes |
|  | Cd80 | nd | nd | nd | nd | **↑3.4 ↓4.2** **↓**1.9 | | r=0.6800  p= 0.0007 |
|  | Cd86 | nd | nd | nd | nd | **↑2.1 ↓3.0 ↓1.7** | | yes |
|  | Ctla4 | nd | nd | nd | nd | **↑6.4 ↓7.5 ↓2.6** | | yes |
|  | Icos | nd | nd | nd | nd | **↑2.3 ↓8.9 ↓3.4** | | yes |
|  | CD28 | nd | nd | nd | nd | **↑1.9 ↓2.3 ↓1.7** | | no |
|  |  |  |  |  |  |  | |  |
| acute phase proteins/ innate immunity | CRP | **↑2.2 ↓3.7 ↓3.3** | yes | nd | nd | nd | | nd |
|  | Haptoglobin | **↑3.0 ↓29.2 ↓21.3** | r=0.5537  p=0.0015 | nd | nd | nd | | nd |
|  | SAP | **↑1.7 ↓1.9 ↓1.7** | yes | nd | nd | nd | | nd |
|  | C3 | nd | nd | nd | nd | **↑8.7 ↓6.5 ↓4.9** | | p = 0.0054  r = 0.5847 |
|  | IL-11 | **↑6.8 ↓10.5 ↓6.0** | yes | nd | nd | nd | | nd |
|  |  |  |  |  |  |  | |  |
| growth and/or differentiation | Csf3 (G-CSF) | nd | nd | same | no | **↑182.2** **↓117.1** **↓1.6** | | yes |
|  | IL-15 | nd | nd | **↑**0.7 **↓**1.0 **↓**5.3 | no | **↑0.3 ↓0.3** **↓**0.6 | | yes  r= -0.8103 |
|  | IL-7 | **↑**1.0 **↓**1.7 **↓2.3** | no | **0.3** **↓**0.6 **↓**0.8 | no | **↑0.4 ↓0.4 ↓**0.6 | | yes  r= -.7979 |
|  | GM-CSF | low | nd | **↑9.0 ↓**4.3 **↓4.0** | no | same | | no |
|  | Ptprc | nd | nd | nd | nd | **↑3.2 ↓4.8 ↓2.2** | | yes |
|  |  |  |  |  |  |  | |  |
| inflammatory cytokines | IFNγ | low | nd | **↑**7.9 **↓92** **↓**2.3 | yes | **↑15.6 ↓38.3** **↓2.8** | | yes |
|  | IL-23p19 | nd | nd | nd | nd | **↑**2.2 **↓**6.3 **↓**1.0 | | yes |
|  | IL-12p35 | nd | nd | nd | nd | **↑36.0 ↓15.0 ↓3.8** | | r=0.6382  p= 0.0019 |
|  | IL-17 | **↑2.8** **↓2.8** **↓2.5** | nd | **↑402 ↓935 ↓402** | yes | **↑151.0 ↓32.8 ↓3.8** | | yes |
|  | IL-18 | **↑1.3 ↓1.9 ↓1.5** | r=0.4374  p= 0.0156 | nd | nd | **↑**0.3 **↓0.2 ↓0.2** | | yes  r= -0.7653 |
|  | IL-1α | **↑0.6** **↓**0.9 **↓2.2** | no | **↑**14.5 **↓**15.0 **↓**4.8 | r=0.4626  p=0.0151 | **↑45.9** **↓115.0** **↓3.2** | | yes |
|  | Il-1β | **↑**1.1 **↓1.3 ↓**1.2 | yes | **↑18.3 ↓42.6** **↓4.9** | yes | **↑52.8 ↓44.2** **↓3.3** | | yes |
|  | IL-6 | **↑2.9 ↓2.9 ↓2.3** | yes | **↑**2.4 **↓4.9** **↓5.9** | no | **↑15.7** **↓35.1** **↓**1.6 | | yes |
|  | TNFα | Low | nd | **↑**21.1 **↓**20.7 **↓**27 | r=0.5358 p=0.0040 | **↑4.9 ↓9.2 ↓2.4** | | yes |
|  |  |  |  |  |  |  | |  |
| anti-inflammatory / Th2 cytokines | IL-10 | **↑**1.2 **↓**1.1 **↓**1.1 | no | **↑**6.2 **↓**9.4 **↓**5.5 | no | **↑3.8 ↓3.9** **↓**1.6 | | no |
|  | IL-13 | nd | nd | **↑**2.0 **↓11.3 ↓26.3** | no | Low | | nd |
|  |  |  |  |  |  |  | |  |
| tissue damage/ repair | MMP-9 | **↑2.0 ↓3.0 ↓1.9** | yes | nd | nd | nd | | nd |
|  | MPO | **↑1.9 ↓2.0 ↓1.8** | yes | nd | nd | nd | | nd |
|  | Nos2 | nd | nd | nd | nd | **↑77.2 ↓17.9 ↓3.0** | | yes |
|  | Vegf | **↑1.3 ↓1.3 ↓1.3** | yes | **↑**3.8 **↓**2.9 **↓**2.2 | no | **↑0.4 ↓0.4** **↓**0.7 | | yes  r= -0.7901 |
|  | TIMP1 | **↑2.9 ↓3.5 ↓2.8** | p = 0.0199  r = 0.4228 | nd | nd | **nd** | | nd |
|  |  |  |  |  |  |  | |  |
| cytokine regulators | Socs1 | nd | nd | nd | nd | **↑3.0 ↓6.6 ↓2.4** | | r= 0.6723  p= 0.0008 |
|  | Socs2 | nd | nd | nd | nd | **↑0.4** **↓0.5** **↓0.6** | | yes  r= -0.7901 |
|  |  |  |  |  |  |  | |  |
| adhesion | Vcam1 | **↑1.3 ↓1.9 ↓1.5** | yes | nd | nd | **↑6.7 ↓7.5 ↓3.6** | | yes |
|  | p-selectin | nd | nd | nd | nd | **↑7.6 ↓7.7** **↓**2.4 | | yes |
|  |  |  |  |  |  |  | |  |
| catabolism | Hmox | nd | nd | nd | nd | **↑2.3 ↓2.1 ↓1.0** | | no |
|  | Pgk1 | nd | nd | nd | nd | **↑1.7 ↓**1.3 **↓**1.2 | | yes |
|  | Gusb3 | nd | nd | nd | nd | **↑1.9 ↓2.1 ↓1.6** | | yes |
|  |  |  |  |  |  |  | |  |
| Transcription factors | Stat1 | nd | nd | nd | nd | **↑2.4 ↓3.6 ↓2.2** | | no |
|  | Tbx21 (Tbet) | nd | nd | nd | nd | **↑1.6 ↓3.0 ↓1.7** | | yes |
|  | Smad3 | nd | nd | nd | nd | **↑0.4 ↓0.5 ↓**0.7 | | p = 0.0022  r = -0.6296 |
|  |  |  |  |  |  |  | |  |
| Misc | Ece1 | nd | nd | nd | nd | **↑0.4 ↓**0.5 **↓**0.6 | | no |
|  | Edn1 (endothelian-1) | nd | nd | nd | nd | **↑0.4 ↓**0.6 **↓**0.5 | | yes  r= -0.7746 |
|  | Ski | nd | nd | nd | nd | **↑0.3 ↓0.6 ↓0.6** | | no |
|  | Bcl2l1 | nd | nd | nd | nd | **↑0.6 ↓0.7 ↓**0.8 | | no |
|  | Tfrc | nd | nd | nd | nd | **↑1.3 ↓**1.4 **↓1.5** | | no |
|  | SGOT | **↑2.2 ↓2.5 ↓1.7** | p = 0.0095  r = 0.4657 |  |  |  | |  |

Numbers shown indicate fold increase in protein and mRNA levels in isotype *Hb* vs. isotype broth, and fold decrease with anti-IL-7Rα M595 500 μg and 50 μg treatments, respectively vs. isotype *Hb*. Bold numbers indicate significant differences (p<0.05). Underlined proteins/mRNA were not significantly altered with disease but were altered with anti-IL-7Rα treatment and/or correlated with disease. **Yes* indicates that protein or mRNA levels significantly correlated with histopathology scores (total histopathology scores for serum and proximal colon scores for colon explant and RNA) at p<0.05 adjusted for 42 multiple comparisons for serum (Bonferroni), 27 multiple comparisons for colon explant, and 90 multiple comparisons for colon mRNA. *Yes* also indicates Spearman’s correlation coefficient r = 0.58 to 0.83. *No* indicates no significant correlation with disease. ^†^Where disease correlations appeared significant despite a >0.05 p-value, unadjusted p-values along with Spearman’s correlation coefficient are shown; all correlations were positive except where negative values are indicated. nd-not done.

Abbreviations

| **Category** | **Abbreviation** | **Name** |
| --- | --- | --- |
| chemokines/ chemokine receptors | Ccl19 (MIP-3β) | C-C motif chemokine ligand 19 (Macrophage inflammatory protein-3 beta) |
|  | Ccl2 (MCP-1) | C-C motif chemokine ligand 2 (Monocyte chemoattractant protein 1) |
|  | Ccl3 (MIP-1α) | C-C motif chemokine ligand 3 (Macrophage inflammatory protein-1 alpha) |
|  | Ccl5 (Rantes) | C-C motif chemokine ligand 5 |
|  | Ccl7 (MCP-3) | C-C motif chemokine ligand 7 (Monocyte chemoattractant protein 3) |
|  | Ccl9 (MIP-1γ) | C-C motif chemokine ligand 9 (Macrophage inflammatory protein-1 gamma) |
|  | Ccl11 (eotaxin) | C-C motif chemokine ligand 11 |
|  | Ccl22 (MDC) | C-C motif chemokine ligand 22 (Macrophage-derived chemokine) |
|  | Ccr2 | C-C motif chemokine receptor 2 |
|  | Ccr7 | C-C motif chemokine receptor 7 |
|  | Cxcl10 (IP-10) | C-X-C motif chemokine 10 (Interferon gamma-induced protein 10) |
|  | Cxcl11 (I-TAC) | C-X-C motif chemokine 11 (Interferon-inducible T-cell alpha) |
|  | Cxcl19 (MIG) | C-X-C motif chemokine 19 (Monokine induced by interferon gamma) |
|  | XCL-1 (Lymphotactin) | C motif chemokine ligand 1 |
|  |  |  |
| leukocyte phenotypic markers | Cd19 | Cluster of Differentiation 19 |
|  | Cd4 | Cluster of Differentiation 4 |
|  | Cd68 | Cluster of Differentiation 68 |
|  | H2-Eα | MHC class II H2-E alpha chain |
|  | IL-2Rα (CD25) | Interleukin 2 receptor alpha |
|  | Tnfrsf18 (Gitr) | Tumor necrosis factor super family 18 (Glucocorticoid-induced TNFR-related protein) |
|  |  |  |
| co-stimulatory molecules | Cd40 | Cluster of Differentiation 40 |
|  | Cd40 ligand | Cluster of Differentiation ligand 40 |
|  | Cd80 | Cluster of Differentiation 80 |
|  | Cd86 | Cluster of Differentiation 86 |
|  | Ctla4 | Cytotoxic T-Lymphocyte Antigen 4 |
|  | Icos | Inducible T-cell costimulator |
|  | CD28 | Cluster of Differentiation 28 |
|  |  |  |
| acute phase proteins/ innate immunity | CRP | C-reactive protein |
|  | Haptoglobin |  |
|  | SAP | Serum amyloid P component |
|  | C3 | Complement component 3 |
|  | IL-11 | Interleukin 11 |
|  |  |  |
| growth and/or differentiation | Csf3 (G-CSF) | Colony-stimulating factor 3 |
|  | IL-15 | Interleukin 15 |
|  | GM-CSF | Granulocyte colony-stimulating factor |
|  | Ptprc | Protein tyrosine phosphatase, receptor type C |
|  |  |  |
| inflammatory cytokines | IFNγ | Interferon gamma |
|  | IL-23p19 | Interleukin 23 subunit 19 |
|  | IL-12p35 | Interleukin 12 subunit 35 |
|  | IL-17 | Interleukin 17 |
|  | IL-18 | Interleukin 18 |
|  | IL-1α | Interleukin 1 alpha |
|  | Il-1β | Interleukin 1 beta |
|  | IL-6 | Interleukin 6 |
|  | TNFα | Tumor necrosis factor alpha |
|  |  |  |
| anti-inflammatory / Th2 cytokines | IL-10 | Interleukin 10 |
|  | IL-13 | Interleukin 13 |
|  |  |  |
| tissue damage/ repair | MMP-9 | Matrix metalloproteinase 9 |
|  | MPO | Myeloperoxidase |
|  | Nos2 | Nitric oxide synthase 2 |
|  | Vegf | Vascular endothelial growth factor |
|  | TIMP1 | tissue inhibitor of metalloproteinase 1 |
|  |  |  |
| cytokine regulators | Socs1 | Suppressor of cytokine signaling 1 |
|  | Socs2 | Suppressor of cytokine signaling 2 |
|  |  |  |
| adhesion | Vcam1 | Vascular cell adhesion protein 1 |
|  |  |  |
| catabolism | Hmox | heme oxygenase |
|  | Pgk1 | Phosphoglycerate kinase 1 |
|  | Gusb3 | β-glucoronidase 3 |
|  |  |  |
| Transcription factors | Stat1 | Signal transducers and activators of transcription 1 |
|  | Tbx21 (Tbet) | T-box transcription factor 21 |
|  | Smad3 | Mothers against decapentaplegic homolog 3 |
|  |  |  |
| Misc | Ece1 | Endothelin converting enzyme 1 |
|  | Edn1 | Eendothelian-1 |
|  | Ski | Sloan-Kettering Institute protooncoprotein |
|  | Bcl2l1 | B-cell lymphoma 2 -like 1 |
|  | Tfrc | Transferrin receptor protein 1 |
